# Integrative and Comprehensive Pancancer Analysis of Regulator of Chromatin Condensation 1 (RCC1)

**DOI:** 10.3390/ijms22147374

**Published:** 2021-07-09

**Authors:** Changwu Wu, Yingjuan Duan, Siming Gong, Sonja Kallendrusch, Nikolas Schopow, Georg Osterhoff

**Affiliations:** 1 Institute of Anatomy, University of Leipzig, Liebigstraße 13, 04103 Leipzig, Germany; wuchangwu93@gmail.com (C.W.); sonja.kallendrusch@medizin.uni-leipzig.de (S.K.); Nikolas.Schopow@medizin.uni-leipzig.de (N.S.); 2 Faculty of Chemistry and Mineralogy, University of Leipzig, 04103 Leipzig, Germany; yingjuan0103@gmail.com; 3 Sarcoma Center, Department of Orthopedics, Trauma and Plastic Surgery, University Hospital Leipzig, 04103 Leipzig, Germany; Georg.Osterhoff@medizin.uni-leipzig.de

**Keywords:** RCC1, pancancer, prognosis, tumor, immune infiltration, enrichment analysis, big data

## Abstract

Regulator of Chromatin Condensation 1 (RCC1) is the only known guanine nucleotide exchange factor that acts on the Ras-like G protein Ran and plays a key role in cell cycle regulation. Although there is growing evidence to support the relationship between RCC1 and cancer, detailed pancancer analyses have not yet been performed. In this genome database study, based on The Cancer Genome Atlas, Genotype-Tissue Expression and Gene Expression Omnibus databases, the potential role of RCC1 in 33 tumors’ entities was explored. The results show that RCC1 is highly expressed in most human malignant neoplasms in contrast to healthy tissues. RCC1 expression is closely related to the prognosis of a broad variety of tumor patients. Enrichment analysis showed that some tumor-related pathways such as “cell cycle” and “RNA transport” were involved in the functional mechanism of RCC1. In particular, the conducted analysis reveals the relation of RCC1 to multiple immune checkpoint genes and suggests that the regulation of RCC1 is closely related to tumor infiltration of cancer-associated fibroblasts and CD8^+^ T cells. Coherent data demonstrate the association of RCC1 with the tumor mutation burden and microsatellite instability in various tumors. These findings provide new insights into the role of RCC1 in oncogenesis and tumor immunology in various tumors and indicate its potential as marker for therapy prognosis and targeted treatment strategies.

## 1. Introduction

Previous studies have shown that Regulator of Chromatin Condensation 1 (RCC1) is the only known guanine nucleotide exchange factor that acts on the nuclear Ras-like G protein Ran in the nucleus [1,2]. It was first identified in BHK cells and was later found to be directly involved in the formation of mitotic spindles, nuclear membrane components, nucleocytoplasmic transport and cell cycle G1/S transition [3,4,5]. In addition, many studies have shown that RCC1 can affect the formation of RanGTP gradient, and its enhanced expression can increase the level of RanGTP, thereby affecting the cell cycle and regulating the DNA damage response [2,6,7,8]. In general, RCC1 is a key cell cycle regulator.

In recent years, more and more evidence has been published on the role of RCC1 in tumor biology. A study by Hsu et al. showed that the expression of RCC1 in lung adenocarcinoma was significantly higher than in normal tissues [9]. A genomewide expression profile analysis of cervical cancer showed that the expression of RCC1 in higher staged tumor tissue (FIGO III) was higher than that of normal cervix [10]. In addition, Riahi et al. identified RCC1 as a candidate breast cancer susceptibility gene through exome sequencing and case–control analysis [11]. These studies indicate that RCC1 may play an important role in the occurrence and development of tumors. Another study showed that the high methylation level of the RCC1 gene in gastric cancer tissue caused the silence of RCC1, which induced the oncogenesis and increased development of invasion depth [12]. This suggests RCC1 to be a tumor suppressor gene in gastric cancer. All the above studies indicate that the expression level and function of RCC1 seem to depend on different tumor types.

Having in mind the key role of RCC1 in cell cycle regulation and evidence related to tumors, it seems necessary to perform a pancancer analysis of RCC1 to clarify its potential role in tumors in general. In this study, various data from The Cancer Genome Atlas (TCGA) database, the Genotype-Tissue Expression (GTEx) database, Gene Expression Omnibus (GEO) database and Clinical Proteomic Tumor Analysis Consortium (CPTAC) dataset were combined, and a variety of tools was used for the first pancancer analysis of RCC1. In the study, we investigated the gene mutation information, expression level, prognostic ability, DNA methylation, protein phosphorylation and gene-immune analysis of RCC1 in different tumors. In addition, we conducted enrichment analysis of related genes to study the potential role and molecular mechanism of RCC1 in tumors. In general, this study aims to understand the role and potential mechanism of RCC1 in a variety of tumors through comprehensive analysis to enable further research. 

## 2. Results

### 2.1. Experiment Setup and Genetic Variation Analysis Results

**Figure 1:**
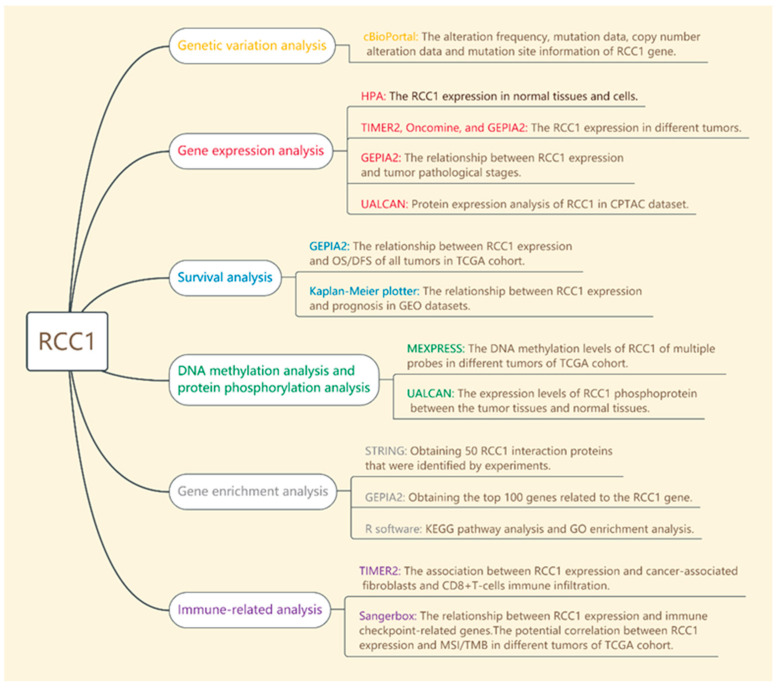
Setup of the integrative and comprehensive Pancancer Analysis of RCC1.

The setup of this study is shown in Figure 1. The following tumor entities were included in this study: adrenocortical carcinoma (ACC), bladder urothelial carcinoma (BLCA), breast invasive carcinoma (BRCA), cervical squamous cell carcinoma and endocervical adenocarcinoma (CESC), cholangiocarcinoma (CHOL), colon adenocarcinoma (COAD), lymphoid neoplasm diffuse large B-cell lymphoma (DLBC), esophageal carcinoma (ESCA), glioblastoma multiforme (GBM), head and neck squamous cell carcinoma (HNSC), kidney renal clear cell carcinoma (KIRC), kidney chromophobe (KICH), kidney renal papillary cell carcinoma (KIRP), liver hepatocellular carcinoma (LIHC), lung adenocarcinoma (LUAD), lung squamous cell carcinoma (LUSC), lower grade glioma (LGG), Acute Myeloid Leukemia (LAML) mesothelioma (MESO), ovarian serous cystadenocarcinoma (OV), prostate adenocarcinoma (PRAD), pheochromocytoma and paraganglioma (PCPG), pancreatic adenocarcinoma (PAAD), rectum adenocarcinoma (READ), stomach adenocarcinoma/gastric cancer (STAD), sarcoma (SARC), skin cutaneous melanoma (SKCM), thyroid carcinoma (THCA), thymoma (THYM), uterine carcinosarcoma (UCS), testicular germ cell tumors (TGCT), uterine corpus endometrial carcinoma (UCEC) and uveal melanoma (UVM).

**Figure 2:**
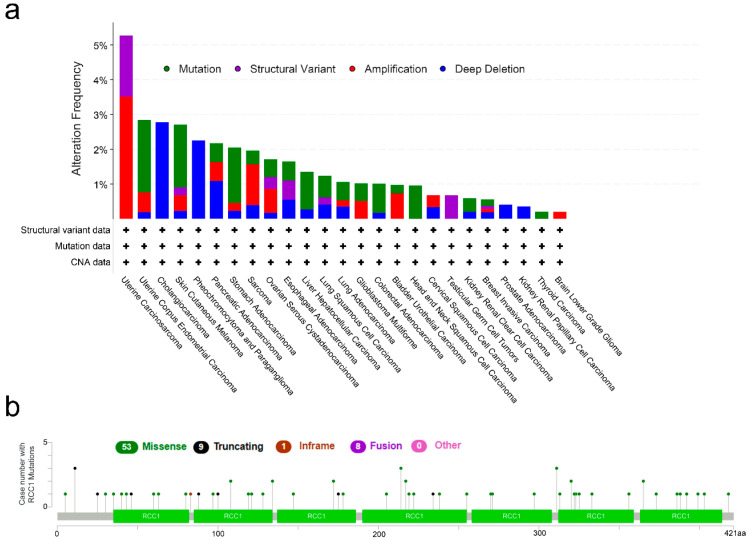
Genetic alternation of RCC1 in different tumors. (**a**) The alteration frequency with mutation type was displayed. (**b**) The mutation types, sites and case number of RCC1 genetic alternation were displayed.

A pancancer analysis of RCC1, a gene localized on the short arm of chromosome 1 (Supplementary Figure S1a), was performed, as well as comparative analysis with corresponding normal tissue. The known regulatory elements of RCC1 are shown in Supplementary Table S1. Further, the RCC1 genetic alternation information of tumors in TCGA cohort was observed. It was found that patients with UCS have the highest frequency of RCC1 genetic alteration (>5%), and the primary type is amplification of copy numbers (Figure 2a). Of note, cholangiocarcinoma and PCPG cases with genetic alternation show a copy number deletion of RCC1 (Figure 2a). As shown in Figure 2b, the types, sites and case number of genetic alternations in RCC1 were displayed, and it was shown that missense mutations were the main type of genetic alternation in RCC1.

### 2.2. Gene Expression and Protein Expression Analysis Results

First, the expression level of RCC1 in normal, healthy tissue and cells was analyzed. As shown in Supplementary Figure S1b, through comprehensive analysis of Human Protein Atlas (HPA), GTEx and Function annotation of the mammalian genome 5 (FANTOM5) datasets, RCC1 showed the highest expression in thyroid gland and testis tissues. However, overall RCC1 showed low RNA tissue specificity in all tissues detected with a normalized expression value >1 in all tissues. Furthermore, based on the combination of HPA, Monaco and Schmiedel datasets, RCC1 also showed low RNA cell type specificity in different blood cells (Supplementary Figure S1c).

**Figure 3:**
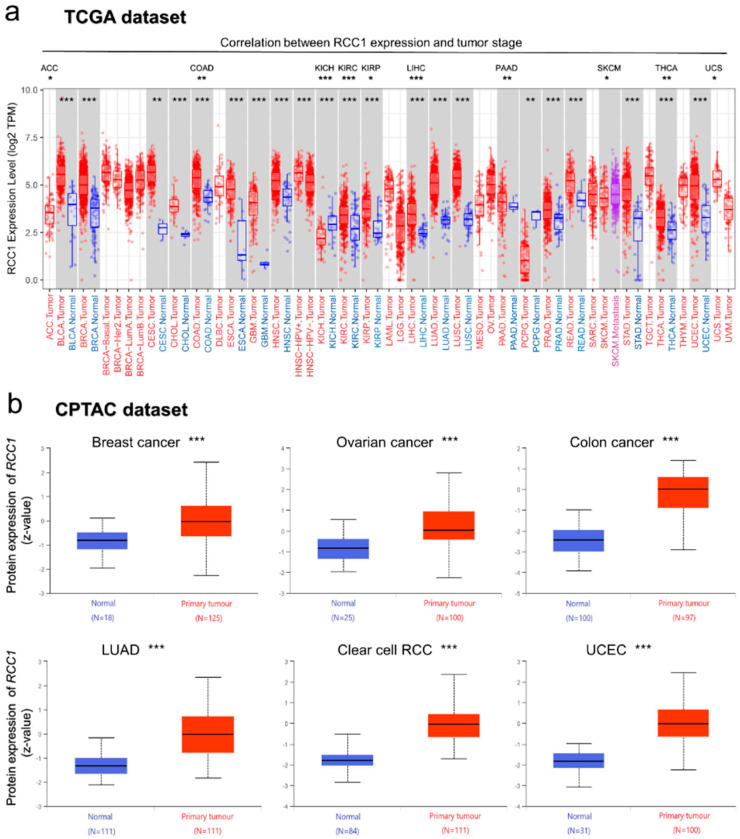
Gene and protein expression levels of RCC1 in different tumors. (**a**) The RCC1 expression of different tumors or Figure 1 expression and main pathological stages. (**b**) The expression level of RCC1 total protein between normal tissue and primary tissue of breast cancer, ovarian cancer, colon cancer, LUAD, clear cell RCC and UCEC. * *p* < 0.05; ** *p* < 0.01; *** *p* < 0.001.

(1) RCC1 expression of different tumors in TCGA dataset was analyzed by use of the Tumor immune estimation resource version 2 (TIMER2) tool. As shown in Figure 3a, the expression of RCC1 in tumor tissues of BLCA, BRCA, CESC, COAD, ESCA, GBM, HNSC, KIRC, KIRP, LICH, LUAD, LUSC, PRAD, READ, STAD, THCA and UCEC is higher than the corresponding normal tissues (all *p* < 0.01). However, in the tumor tissues of KICH (*p* < 0.001) and PCPG (*p* < 0.01), the expression level of RCC1 is lower than the corresponding normal tissues. Further, it was found that the expression level of RCC1 was related to the tumor pathological stages of KIRP, LIHC, ACC, COAD, KICH, KIRC, PAAD, UCS, THCA and SKCM (Figure 3a, Supplementary Figure S2a, all *p* < 0.05). No correlation was found in other tumors, such as BLCA (Supplementary Figure S2b).

Based on the combination of TCGA and GTEx datasets, a supplementary analysis of the expression of RCC1 in tumor tissues was performed. It was confirmed that the expression of RCC1 in BRCA, CESC, DLBC, GBM, LGG, PAAD, THYM and UCS tumor tissues was higher than the corresponding normal tissues (Supplementary Figure S3a, all *p* < 0.01). No significant difference in RCC1 expression was found in ACC, OV, SKCM and TGCT (Supplementary Figure S3b). At the same time, the pooled analysis of different studies in the Oncomine database confirmed that compared with normal tissues, RCC1 is highly expressed in lung cancer, colorectal cancer, breast cancer, bladder cancer, brain cancer, sarcoma, prostate cancer, and leukemia (Supplementary Figure S4).

Analysis in the CPTAC dataset showed that the expression of RCC1 total protein in primary tumor tissues of breast cancer, ovarian cancer, colon cancer, LUAD, RCC and UCEC was higher than that in normal tissues (Figure 3b).

### 2.3. Survival Analysis Results

**Figure 4:**
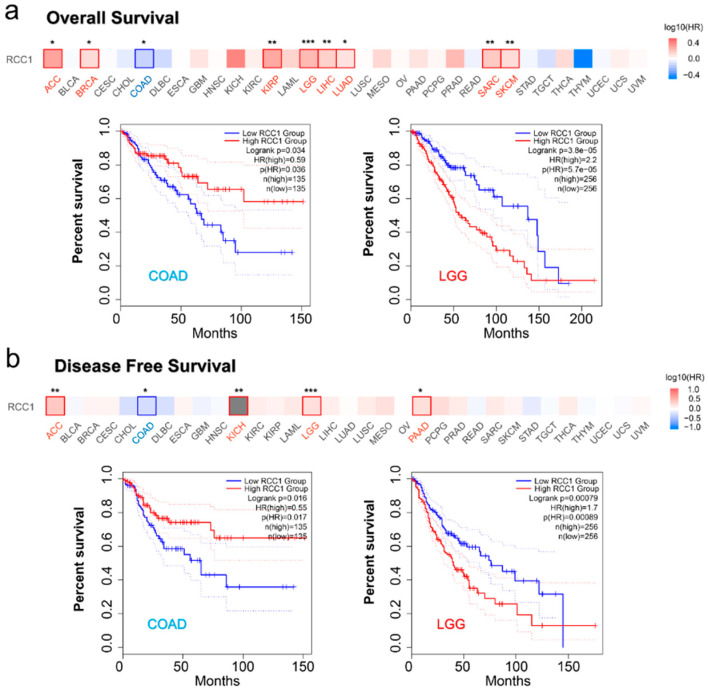
Correlation between RCC1 gene expression and survival prognosis of tumors in TCGA dataset. (**a**) The correlation between RCC1 expression and overall survival of different tumor patients. (**b**) The correlation between RCC1 expression and disease-free survival of different tumor patients. The survival maps and Kaplan–Meier curves of LGG and COAD were given. * *p* < 0.05; ** *p* < 0.01; *** *p* < 0.001.

The TCGA dataset was divided into a group with high expression and a group with low expression of RCC1 to explore the correlation between RCC1 expression and the prognosis of different tumor patients. It was found that the high expression of RCC1 was associated with the poor overall survival (OS) of ACC, BRCA, KIRP, LGG, LIHC, LUAD, SARC and SKCM, and high RCC1 expression was an adverse risk factor for OS. Among all tumor entities, LGG showed the greatest significance (Figure 4a, all *p* < 0.05). High RCC1 expression is related to poor disease-free survival (DFS) of ACC, KICH, LGG and PAAD (Figure 4b, all *p* < 0.05). However, in COAD, the high RCC1 expression group had better OS and DFS (Figure 4a,b, all *p* < 0.05).

Kaplan–Meier survival analyses showed that high expression of RCC1 is associated with poor OS of KIRC (*p* = 0.00012, Supplementary Figure S5a) and PAAD (*p* = 0.0076). In contrast, a low expression of RCC1 is associated with poor OS of ESCA (*p* = 0.00087) and READ (*p* = 0.0023). In addition, the high expression of RCC1 is also associated with poor disease-free survival (DFS) of sarcoma and better DFS of bladder cancer (Supplementary Figure S5b, all *p* < 0.05). In STAD, the low expression of RCC1 is associated with poor OS, first progression (FP) and post progression survival (PPS) (Supplementary Figure S5c, all *p* < 0.001). In contrast, the high expression of RCC1 is associated with poor OS, FP and PPS for lung cancer (Supplementary Figure S5d, all *p* < 0.05). Additionally, the high expression of RCC1 is also related to poor OS, relapse-free survival (RFS), distant metastasis-free survival (DMFS) and PPS in breast cancer and poor OS, RFS, disease-specific survival (DSS) and progress-free survival (PFS) in liver cancer (Supplementary Figure S5e,f, all *p* < 0.05). However, in ovarian cancer, a high expression of RCC1 was only found to be associated with poor PPS (*p* = 0.0083), and no correlation between RCC1 expression and OS and PFS was detected (Supplementary Figure S5g). The above data indicated that the relationship between the expression of RCC1 and the prognosis of tumor patients depends on different tumor types.

### 2.4. DNA Methylation Analysis and Protein Phosphorylation Analysis Results

**Figure 5:**
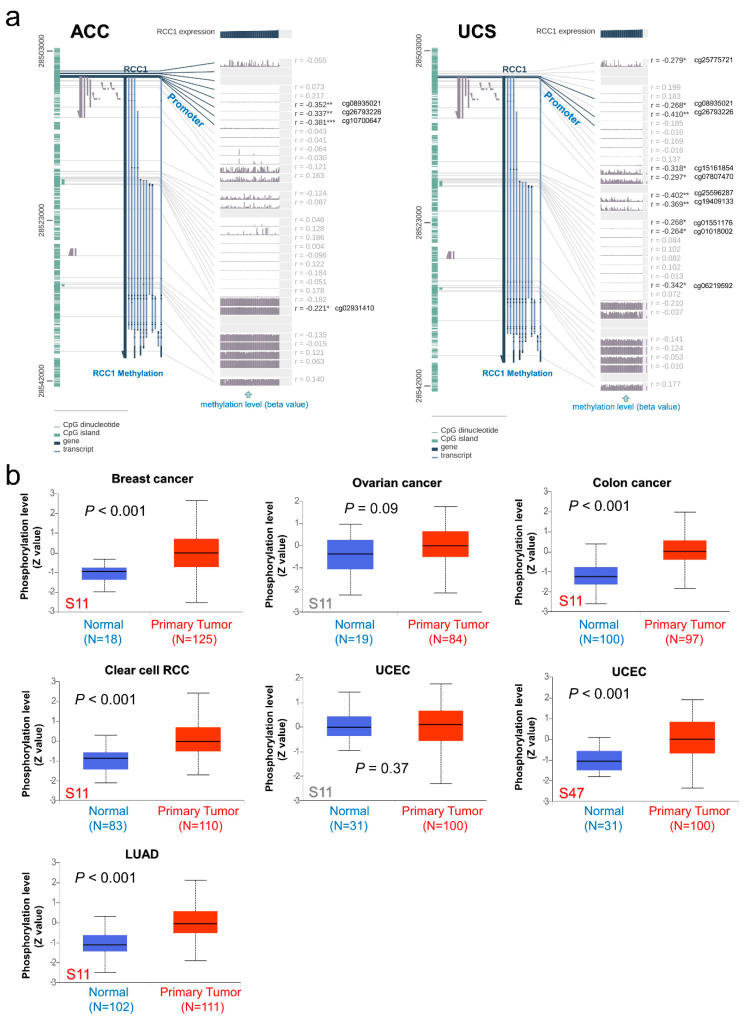
DNA methylation analysis and protein phosphorylation analysis of RCC1. (**a**) Association between RCC1 DNA methylation and gene expression for the ACC and UCS cases of TCGA. * *p* < 0.05; ** *p* < 0.01; *** *p* < 0.001. (**b**) The expression level of RCC1 phosphoprotein (S11 and S47 sites) between normal tissue and primary tissue of selected tumors.

Using MEXPRESS tool, the potential relationship between RCC1 DNA methylation and tumor pathogenesis in TCGA cohort tumors was explored. In ACC, it was found that RCC1 DNA methylation was significantly negatively correlated with RCC1 gene expression at multiple probes in the promoter region (Figure 5a, all *p* < 0.01). Additionally, in UCS, not only at the multiple probes of promoter region but also at the multiple probes of non-promoter region, a significant negative correlation between RCC1 DNA methylation and gene expression was observed (Figure 5a, all *p* < 0.05). Correlation of RCC1 gene expression with RCC1 DNA methylation in multiple probes of other tumors is shown in Supplementary Table S2. Based on the CPTAC dataset, the differences in the phosphorylation levels of RCC1 between the six tumors (breast cancer, colon cancer, clear cell RCC, LUAD, ovarian cancer, UCEC) and the corresponding normal tissues were also analyzed. Compared with normal tissues, the S11 locus of RCC1 showed higher phosphorylation levels in breast cancer (*p* < 0.001), colon cancer (*p* < 0.001), clear cell RCC (*p* < 0.001) and LUAD (*p* < 0.001), but not significantly in ovarian cancer (*p* = 0.09) and UCEC (*p* = 0.37) (Figure 5b). In addition, the S47 locus of RCC1 has a higher phosphorylation level in the primary tumor tissue of UCEC than in normal tissue (Figure 5b, *p* < 0.001).

### 2.5. Gene Enrichment Analysis Results

**Figure 6:**
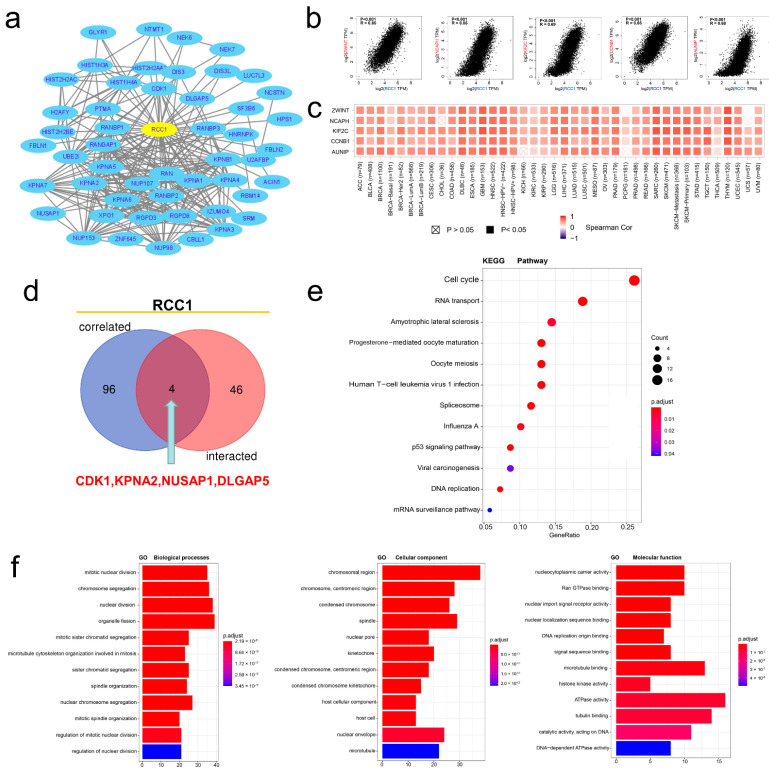
RCC1-related gene enrichment analysis. (**a**) A protein–protein interaction network of 50 experimentally verified RCC1-interacted proteins. (**b**) The top 100 genes related to RCC1 expression were obtained, and the expression correlation between SND1 and the top five genes was shown, including ZWINT, NCAPH, KIF2C, CCNB1 and AUNIP. (**c**) The corresponding heatmap data in the detailed tumor types were displayed. (**d**) An intersection analysis of the RCC1-interacted and RCC1-correlated genes was conducted and four genes were obtained, including CDK1, KPNA2, NUSAP1 and DLGAP5. (**e**) Based on the RCC1-interacted and RCC1-correlated genes, KEGG pathway analysis was performed. (**f**) Based on the SND1-binding and interacted genes, GO analysis was performed.

In order to further explore the potential mechanism of RCC1 in oncogenesis and development, protein–protein interaction (PPI) analysis and RCC1-correlated gene analysis were carried out based on STRING tool and Gene expression profiling interactive analysis version 2 (GEPIA2) tool. As the PPI network shown in Figure 6a, a total of 50 proteins that interacted with RCC1 have been obtained, which was supported by experimental evidence. In addition, the top 100 genes related to RCC1 expression were obtained, among which the top five genes were ZW10 interacting kinetochore protein (ZWINT) (R = 0.65), non-SMC condensin I complex subunit H (NCAPH) (R = 0.65), kinesin family member 2C (KIF2C) (R = 0.69), cyclin B1 (CCNB1) (R = 0.65) and aurora kinase A and ninein interacting protein (AUNIP) (R = 0.68) (Figure 6b, all *p* < 0.001). The heat map shows that in most tumor types, the expression of RCC1 was positively correlated with these five genes (Figure 6c). Additionally, four common members were obtained by cross-analyzing the above two groups, namely cyclin dependent kinase 1 (CDK1), karyopherin subunit alpha 2 (KPNA2), nucleolar and spindle associated protein 1 (NUSAP1) and DLG associated protein 5 (DLGAP5) (Figure 6d). By combining the two sets of data, Kyoto encyclopedia of genes and genomes (KEGG) pathway analysis and Gene ontology (GO) enrichment analysis were performed. KEGG pathway analysis indicated that RCC1 may be involved in oncogenesis through the “cell cycle”, “RNA transport”, “p53 signaling pathway”, “DNA replication” and “mRNA surveillance pathway” pathways (Figure 6e). The GO enrichment analysis suggested that these genes were mainly related to biological processes such as “mitotic nuclear division” and “chromosome segregation”, cellular components such as “chromosomal region” and “chromosome, centromeric region” and molecular function such as “cytoplasmic carrier activity” and “Ran GTPase binding” (Figure 6f).

### 2.6. Immune-Related Analysis Results

**Figure 7:**
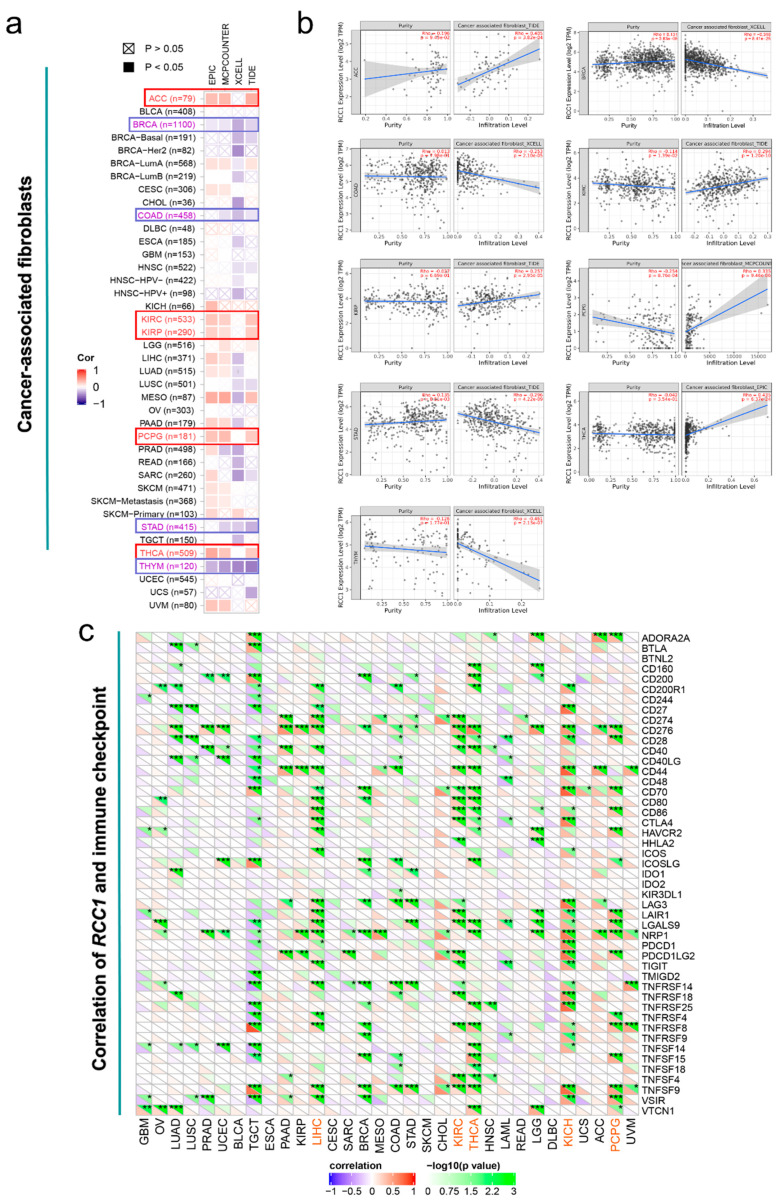
Immune-related analysis of RCC1. (**a**) Association between RCC1 expression and immune infiltration of cancer-associated fibroblasts in different tumors. (**b**) The scatter plots of cancer-associated fibroblasts immune infiltration in different tumors generated based on a certain algorithm. (**c**) Association between RCC1 expression and immune checkpoint genes expression in different tumors. * *p* < 0.05; ** *p* < 0.01; *** *p* < 0.001.

Based on the EPIC, MCPCOUNTER, XCELL, TIDE, TIMER, CIBERSORT, CIBERSORT-ABS and QUANTISEQ algorithms, the correlation between the expression of RCC1 and cancer-associated fibroblasts (CAF) and the infiltration level of CD8^+^ T-cells were estimated in different TCGA cohort tumors. Correlations with the same trend in most algorithms were considered to be credible. As shown in Figure 7a, the expression of RCC1 and CAF are significantly positively correlated in ACC, KIRC, KIRP, PCPG and THCA, but negatively correlated in BRCA, COAD, STAD and THYM (all *p* < 0.05). The scatter plots generated based on the algorithm with the best significance are shown in Figure 7b. In addition, it was also observed that the expression of RCC1 was positively correlated with the tumor infiltration level of CD8^+^ T-cells in THYM and UVM (Supplementary Figure S6, all *p* < 0.05). The immune checkpoint analysis, based on the Sangerbox tool, showed that the expression of RCC1 in LIHC, KIRC, THCA, KICH and PCPG was positively correlated with most of immune checkpoint genes, especially CD276 (also known as B7-H3) and neuropilin-1 (NRP1), and the specific results are shown in Figure 7c. Furthermore, we also detected a relationship between CD276 and NRP1 expression and CD8^+^ T cell infiltration (Supplementary Figure S7). In most tumors, except UVM, the higher the expression of CD276, the higher the level of CD8^+^ T cell infiltration. Moreover, the high expression of NRP1 is related to the high CD8^+^ T cell infiltration in PAAD.

**Figure 8:**
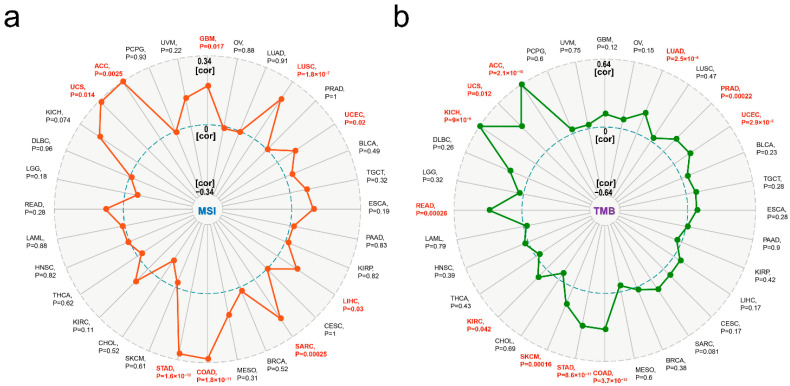
Correlation between RCC1 and microsatellite instability/tumor mutational burden in different tumors of TCGA dataset. (**a**) MSI. (**b**) TMB.

Furthermore, the relationship between the expression of RCC1 and microsatellite instability (MSI)/tumor mutation burden (TMB) of the tumors in TCGA cohort was analyzed. It was found that in ACC, GBM, LUSC, UCEC, LIHC, SARC, COAD, STAD and UCS, the expression of RCC1 was all positively correlated with MSI (Figure 8a, all *p* < 0.05) and the expression of RCC1 in ACC, LUAD, PRAD, UCEC, COAD, STAD, SKCM, KIRC, READ, KICH and UCS was positively correlated with TMB (Figure 8b, all *p* < 0.05). The above results indicate that RCC1 is closely related to the immune status of tumors.

## 3. Discussion

RCC1 regulates the cell cycle and was shown to be related to DNA damage and tumor development [13,14,15]. In this study, numerous genetic analysis tools, such as GEPIA2, TIMER2 and UALCAN, were applied to analyze more than 10,000 samples retrieved from TCGA, GEO, GTEx and CPTAC datasets on the expression of RCC1 gene in 33 tumor entities. These datasets were analyzed for genetic variation, altered gene expression, survival analysis, DNA methylation, protein phosphorylation and immune infiltration. Additionally, an enrichment analysis was conducted to relate potential mechanisms to RCC1 expression. The various results involving this study were summarized in Supplementary Table S3.

Compared with normal tissues, in most tumors the transcription and protein levels of RCC1 were increased and high expression of RCC1 was associated with significantly reduced prognoses (e.g., in BRCA and KIRC). This observation suggests that RCC1 has a cancer-promoting effect in these tumors. Still, the expression and function of RCC1 depend on the different tumor types. On the one hand, as RCC1 methylation downregulates RCC1 expression and promotes the oncogenesis and invasion of STAD, RCC1 shows the potential to regulate DNA replication and inhibit tumors [12]. On the other hand, RCC1 promotes oncogenesis and drug resistance in a variety of tumors (e.g., in LUAD, OV and others) [9,10,11,16].

Here, lung cancer was divided into two different pathological subtypes, namely LUAD and LUSC. In the TCGA cohort, although the expression of RCC1 in LUAD and LUSC is higher than that in normal tissue, the high expression of RCC1 is only related to poor OS in patients with LUAD. Additionally, subsequent analyses based on the GEO cohort confirmed that RCC1 was associated with poor OS, FP, and PPS of LUAD (Supplementary Figure S5d) and not of LUSC (Supplementary Figure S8). This indicates that RCC1 may have different prognostic effects in variable lung cancer subtypes, but further research is needed to clarify its relevance.

The analysis of digestive tract lumen organ tumors (ESCA, COAD, READ and STAD) showed specific mechanisms that differed from those of other tumors. Although the expression of RCC1 in these four kinds of digestive tract tumors is higher than that in normal tissues, the high expression of RCC1 is correlated with a better prognosis and the expression of RCC1 in COAD is negatively correlated with tumor stage. This can be seen as being unlike many other tumors and therefore seems to be contradictory. Subsequent immune-related analyses found that the expression of RCC1 in COAD and STAD was negatively correlated with the immune infiltration of CAF. The high expression of RCC1 is related to a favorable prognosis of COAD and STAD and can be related to immune infiltration. This correlation is worth further investigations. Thus far, there is no literature on the prognostic role of RCC1 in gastrointestinal tumors. Therefore, our results suggest a new alternative prognostic biomarker for tumors of the gastrointestinal tract.

In this study, RCC1-interacted proteins and RCC1-related genes in all tumors were integrated, and four common genes (CDK1, KPNA2, NUSAP1, DLGAP5) were obtained by cross-analysis between RCC1-interacted group and RCC1-related group. Among them, the correlation between RCC1 and CDK1 has been verified. Qiao et al. confirmed that RCC1 regulates the expression of CDK1 through transcription factor E2F1 (especially nuclear E2F1) to promote the transition of G_1_ to S phase of the cell cycle [17]. The potential regulatory relationship between RCC1 and KPNA2, NUSAP1 and DLGAP5 is still unclear, and further studies on them may help to understand the mechanism of RCC1 in tumors. Furthermore, a series of enrichment analyses was performed on the above two groups. Not surprisingly, RCC1-related and RCC1-interacted genes are mainly involved in known pathways linked to the cell cycle, DNA replication and RNA transport [5,6,13,18]. Notably, enrichment analysis showed that these genes were associated with the p53 signaling pathway, and there was currently no report on the potential relationship between RCC1 and this pathway.

The current consensus is that somatic mutations play a vital role in the development of tumors [19,20]. However, tumor-specific antigens (=neoantigens) based on somatic mutations have also shown new paths in cancer treatment [21]. Particularly, cancer vaccines targeting neoantigens have obtained considerable benefits in mouse models of various tumors such as melanoma and pancreatic cancer [22,23]. Designing vaccines based on patient-specific gene mutations may be the direction of individualized tumor treatment in the future. Our results show that RCC1 has frequent mutations in multiple tumors including UCEC, SKCM, STAD and LIHC. For personalized tumor vaccines, every mutated gene that may encode tumor-specific antigens in an individual patient could be a potential target. Although experimental studies still need to confirm its potential as a neoantigen, these data suggest RCC1 as a new target for cancer vaccines especially in UCEC due to the most frequent mutations in UCEC.

The tumor microenvironment (TME), including tumor-infiltrating immune cells, stromal cells and the vascular system, usually blocks the effective immune response of the host [24,25,26]. Other studies have shown that tumor-infiltrating immune cells are closely related to oncogenesis, progression and metastasis [26,27]. CAF, as one of the most abundant components of the TME stroma, are believed to participate in the functional regulation of tumor-infiltrating immune cells in promoting tumors and are closely related to the immune evasion of tumor cells. [27,28,29,30]. The latest research shows that CAF-targeted therapy is expected to provide new ways to overcome tumors by reducing immunosuppressive events and reshaping TME [30]. CD8^+^ T cells are the key factor in anticancer immunity [31,32,33]. However, tumor-infiltrating CD8^+^ T cells often present themselves in an “exhaustion” state due to long-term exposure to persistent antigens and inflammation in the absence of co-stimulatory signals and lack of nutrients and oxygen in the TME [31,32,34]. Using a variety of immune deconvolution methods, for the first time to our knowledge, this study observed the relationship between RCC1 and CAF together with tumor-infiltrating CD8^+^ T cells. The positive correlation between RCC1 expression and CAF infiltration in ACC and KIRC may be part of the reason why high RCC1 expression is related to a worse prognosis of ACC and KIRC. In THYM, the expression of RCC1 is not only negatively correlated with CAF, but also positively correlated with CD8^+^ T cell tumor infiltration, which may imply that targeted drugs designed for RCC1 can effectively assist immunotherapy against THYM.

The discovery of immune checkpoints, including cytotoxic T lymphocyte-associated protein-4 (CTLA-4) and programmed cell death protein-1 (PD-1, also known as PDCD1), was crucial to the development of cancer immunotherapy and has brought revolutionary changes [35,36]. Immune checkpoint inhibitors targeting CTLA-4 and PD-1 have been applied therapeutically and have obtained impressive clinical benefits [36,37]. Therefore, in addition to immune infiltration, this study also analyzed the relationship between RCC1 expression and the expression of 47 common immune checkpoint genes and found that they are related in a variety of tumors, including KIRC and KICH. In KIRC and KICH, the expression of RCC1 was positively correlated with the expression of both PD-1 and/or CTLA4. In view of the current research showing that the combined use of PD-1 inhibitors (nivolumab) and CTLA4 inhibitors (ipilimumab) brings significant survival advantages for patients with renal cell carcinoma [38], the strong correlation between RCC1 and the two checkpoints indicates that it may be a powerful immunotherapy marker for KICH and KIRC. A recently conducted study in non-small cell lung cancer pointed out that downregulation of RCC1 can increase the sensitivity of immunotherapy by upregulating PD-L1 through the p27^kip1^/CDK4 axis [39]. In addition, most RCC1 positive tumors correlated with CD276 and NRP1 (Figure 7c). Current research shows that CD276 expression is elevated in most tumors, including melanoma and lung cancer, and is closely related to the inhibition of T-cell response and immune evasion of tumors [40,41]. CD276 has become an attractive target for cancer immunotherapy. The use of monoclonal antibodies against CD276 has shown encouraging results in a phase I clinical trial [42]. Based on the clinical success of immune checkpoint inhibitors (ipilimumab and nivolumab), RCC1 may play a potential role in predicting the therapeutic response of CD276 inhibitors developed for clinical use in the future and has potential clinical significance. NRP1 is expressed on human plasmacytoid dendritic cells and may enhance T regulatory cells’ tumor infiltration to promote immunosuppression [43,44,45]. In addition, the research on NRP1 monoclonal antibodies and cell penetrating peptides highlights it as a promising new target for cancer therapy [46,47,48]. Considering the importance of NRP1 in tumor development and its relevance to RCC1 in a variety of tumors, combined with RCC1 involved in CAF and the tumor infiltration of CD8^+^ T cells, we believe that RCC1 might be a potential new tumor target involved in the immunosuppressive function of NRP1. Although no regulatory relationships between RCC1 and CD276 as well as NRP1 have been reported in the literature, a potential regulatory network was identified based on the GeneMANIA 3.6.0 (http://www.genemania.org, accessed on 20 June 2021) tool (Supplementary Figure S9). This finding further supports our results.

Recent evidence indicates that TMB is a potential biomarker for predicting the response to immune checkpoint blockade [49,50,51,52]. This study found that there is a positive correlation between RCC1 expression and TMB in tumors such as KIRC and KICH, combined with the correlation between RCC1 and immune checkpoint genes, which seems to suggest a potential relationship among the three. In addition, it was shown that MSI, together with TMB and PD-1/CTLA4 expression, can serve as a predictive biomarker for immunotherapy [53,54,55,56]. The present study clarified that the expression of RCC1 is positively correlated with MSI in a variety of tumors including GBM, ACC and COAD. Therefore, RCC1 may also have the potential to serve as a predictive biomarker for immunotherapy.

Although our study aggregates a large amount of clinical data from different databases, there are limitations. First of all, even if bioinformatics big data analysis provides us with meaningful insights into RCC1 in oncogenesis and tumor development, the data of in vivo and in vitro experiments on the effect of RCC1 alterations in CAF, CD8^+^ T cell infiltration and immunological function are still scarce and should be intensified. Secondly, some tumors are limited in sample size due to their incidence; a larger sample size is needed to verify the role of RCC1 in these tumors.

Taken together, this study presents the findings of a comprehensive pancancer analysis of RCC1, which was the first ever investigation of the statistical correlation between RCC1 expression and clinical prognosis, DNA methylation, protein phosphorylation, immune infiltration, TMB and MSI in a variety of tumors. Our research found that RCC1 has tumor-promoting effects in most tumors including BRCA, KIRC, LGG, and LUAD, while it seems to have tumor-suppressing effects in ESCA, COAD, READ and STAD. This study provides important insights on clarifying the role of RCC1 in oncogenesis. In particular, it shows a link between RCC1 and p53 for the first time. In addition, it also advances the understanding of the impact of RCC1 on tumor immunology with significant correlation to CAF and tumor-infiltrating CD8^+^ cells. Our findings therefore suggest RCC1 as a new prognostic biomarker and therapeutic target for immunotherapy in different tumor types.

## 4. Materials and Methods

### 4.1. Genetic Variation Analysis

Using the cBioPortal tool (https://www.cbioportal.org/, accessed on 20 June 2021) [57], the genetic variation of RCC1 gene in tumors was analyzed. We selected the “TCGA Pan Cancer Atlas Studies” module in cBioPortal, and then entered the RCC1 gene to query the cancer types summary, and obtained the alteration frequency, mutation data and copy number alteration (CNA) data. Furthermore, the mutation site information of RCC1 gene was obtained in the “Mutation” module.

### 4.2. Gene Expression and Protein Expression Analysis Results

The expression data of RCC1 in different normal tissues and cells were obtained through the HPA database (https://www.proteinatlas.org/humanproteome/pathology, accessed on 20 January 2021). “Low specificity” was defined by “NX (Normalized expression) ≥1 in at least one tissue/region/cell type but not elevated in any tissue/region/cell type”.

The expression difference of RCC1 between tumor and adjacent normal tissues in the TCGA dataset was obtained by TIMER2 tool (http://timer.cistrome.org/, accessed on 25 January 2021). Since there are certain tumor tissues without adjacent normal tissues or the normal tissues are highly limited, supplementary analyses were conducted through the online tool GEPIA2 (http://gepia2.cancer-pku.cn/#analysis, accessed on 20 January 2021) [58]. In the “Expression Analysis” module of GEPIA2, the parameters were set to “log2FC cutoff = 1, *p*-value cutoff = 0.01”, and the box plots of the difference between the tumor tissues and the corresponding normal tissues in the GTEx database were obtained. Furthermore, the difference of RCC1 expression between certain tumor tissues and adjacent normal tissues was analyzed in the Oncomine database (https://www.oncomine.org/resource/main.html, accessed on 20 January 2021).

In addition, through the “Expression Analysis-Stage Plot” module of GEPIA2, we obtained the difference of RCC1 expression among different pathological stages of different tumors in TCGA dataset. The “log2(TPM + 1)” was used for log-scale in violin plots.

Protein expression analysis was performed in CPTAC dataset by UALCAN tool (http://ualcan.path.uab.edu/analysis-prot.html, accessed on 20 January 2021) [59]. We selected six available tumor datasets, including breast cancer, ovarian cancer, colon cancer, LUAD, RCC and UCEC, and compared the difference of total protein expression of RCC1 between tumor tissues and normal tissues.

### 4.3. Survival Analysis

The OS and DFS of all tumors in the TCGA cohort were analyzed through the “survival analysis” module of GEPIA2. Based on the median value of RCC1 expression, patients were divided into high expression groups and low expression groups. Survival maps and Kaplan–Meier survival curves were obtained, log-rank *p* value and hazard ratio (HR) were calculated. For further supplementary analysis, the Kaplan–Meier plotter tool (http://kmplot.com/analysis/, accessed on 20 January 2021) was also used to summarize different GEO datasets to analyze the OS, RFS, FP, PPS, PFS, DMFS and DSS of different tumors. By setting the parameter to “autoselect best cutoff”, patients with different tumors were divided into two groups to generate Kaplan–Meier survival curves and calculated log-rank *p* value, HR and 95% confidence intervals.

### 4.4. DNA Methylation Analysis and Protein Phosphorylation Analysis

The MEXPRESS tool (https://mexpress.be, accessed on 28 January 2021) was used to explore the DNA methylation levels of RCC1 of multiple probes in different tumors of TCGA cohort. The promoter regions probes were highlighted. The beta value of each sample, Pearson correlation coefficient (r) value and *p* value were obtained. In addition, we also analyzed the expression levels of RCC1 phosphoprotein between the tumor tissues and normal tissues of six available tumor -data in the CPTAC dataset through the UALCAN portal.

### 4.5. Gene Enrichment Analysis

Using the STRING tool (https://string-db.org/, accessed on 10 March 2021), PPI analysis of RCC1 gene was performed. We obtained no more than 50 RCC1 interaction proteins that were identified by experiments, and used Cytoscape software to visualize the PPI network. In addition, based on all tumor tissues and normal tissues in TCGA cohort, we also used the “Similar Genes Detection” module of GEPIA2 to obtain the top 100 genes related to the RCC1 gene. For the top 5 genes in correlation, Pearson correlation analysis with RCC1 in the “Correlation Analysis” module of GEPIA2 was performed to obtain the *p*-values, the correlation coefficient values and dot plots. Furthermore, Spearman’s correlation test was performed on these five genes in the “Gene_Corr” module of the TIMER2 tool and *p*-values, partial correlation values and heatmap were obtained.

Using the Draw Venn Diagram tool (http://bioinformatics.psb.ugent.be/webtools/Venn/, accessed on 10 March 2021) [60], RCC1-interacted genes and RCC1-correlated genes were cross-analyzed, and a Venn diagram was generated. In addition, combining the two sets of data, R package “clusterProfiler” in R software (Version 4.0.3, R Foundation for Statistical Computing, Vienna, Austria) was also used to perform KEGG pathway analysis and GO enrichment analysis. Adjusted *p*-values were obtained from multiple hypothesis testing using the Benjamini–Hochberg method, *p*.adjust < 0.05 was considered statistically significant.

### 4.6. Immune-Related Analysis

The TIMER2 tool was used to explore the association between RCC1 expression and immune infiltration of all tumors in the TCGA cohort. In TIMER2, the “immune” module was selected, CAF and CD8^+^ T-cells were further selected, and EPIC, MCPCOUNTER, XCELL, TIDE, TIMER, CIBERSORT, CIBERSORT-ABS and QUANTISEQ algorithms were used to estimate immune infiltration. Spearman’s correlation test after purity adjustment was used to calculate *p* values and partial correlation values. In addition, through the Sangerbox tool, the relationship between RCC1 expression and many immune checkpoint-related proteins was explored. Moreover, the Sangerbox tool (http://sangerbox.com/Tool, accessed on 20 March 2021) was used to explore the potential correlation between RCC1 expression and MSI or TMB in different tumors of TCGA cohort [61]. Spearman’s rank correlation test was also performed to obtain *p*-value and partial correlation (cor) value.

## Data Availability

The data provided in this study can be obtained in the method section of this manuscript.

## Supplementary Materials

The following are available online at https://www.mdpi.com/article/10.3390/ijms22147374/s1.
