# Supplementary material for: Integrative and Comprehensive Pancancer Analysis of Regulator of Chromatin Condensation 1 (RCC1)
Source: Int J Mol Sci. 2021 Jul 9;22(14):7374. doi: 10.3390/ijms22147374 (PMC8305170; doi:10.3390/ijms22147374)
Supplement: Supplementary file 1 [file ijms-22-07374-s001.zip › Supplementary Materials.pdf]

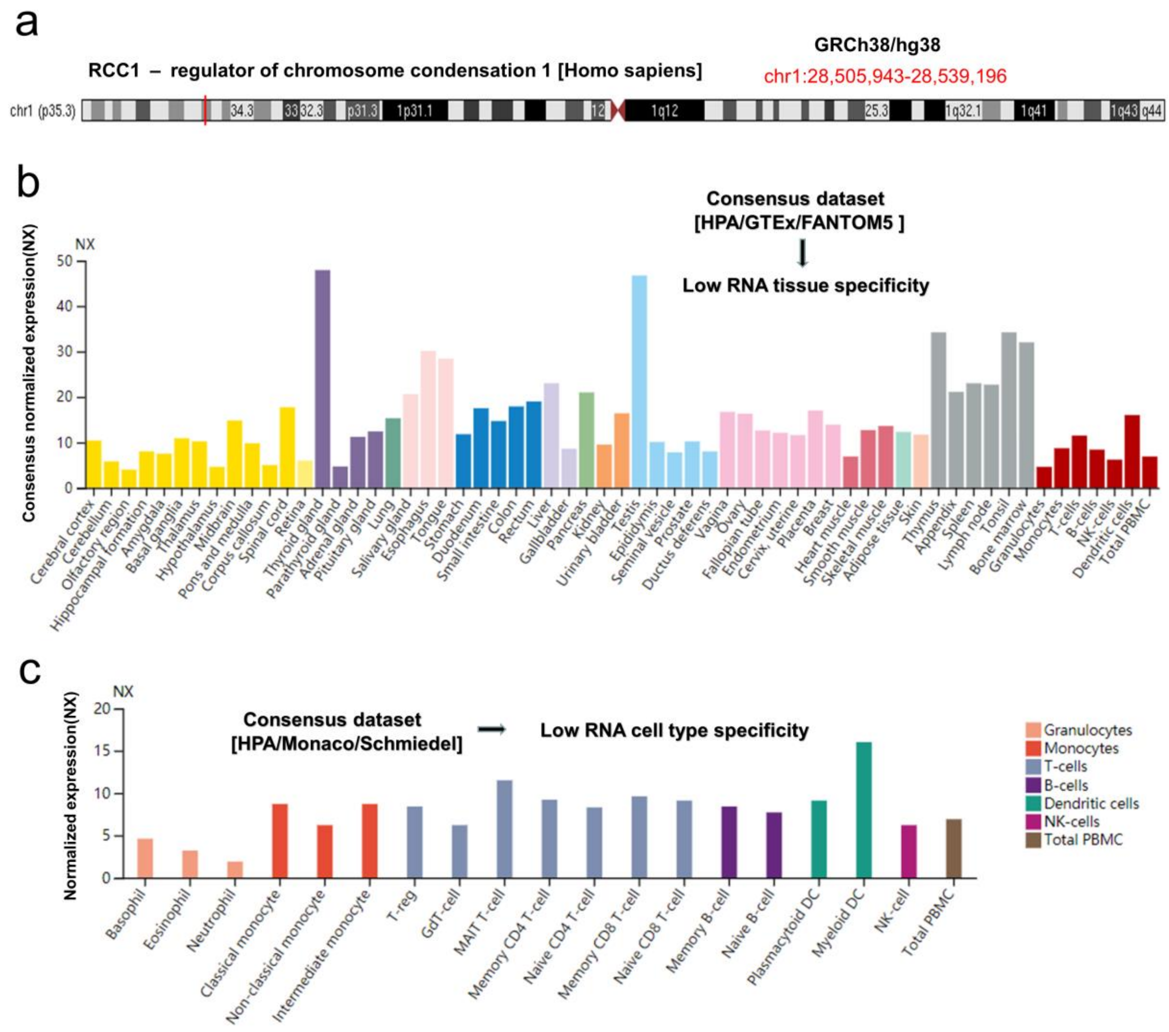

Supplementary Figure S1. Expression level of RCC1 in different normal tissues and blood cells. (a) Genomic location of human RCC1. (b) RCC1 gene in different normal tissues using the consensus datasets of HPA, GTEx and FANTOM5. (c) RCC1 gene in different normal blood cells using the consensus datasets of HPA, Monaco and Schmiedel.

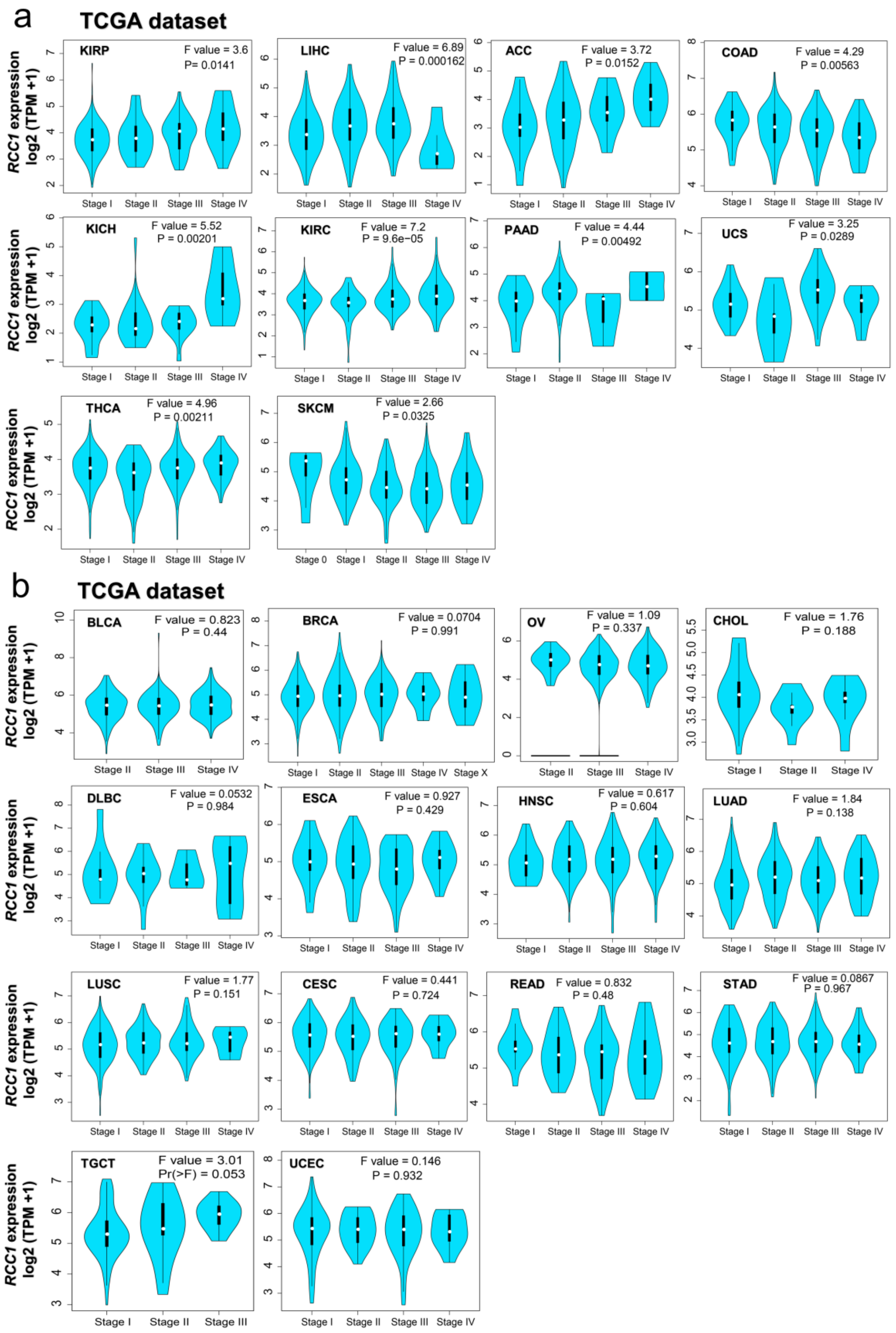

Supplementary Figure S2. The correlation between RCC1 expression and main pathological stages in TCGA dataset. (a) The expression of RCC1 gene were analyzed by the main pathological stages of KIRP, LIHC, ACC, COAD, KICH, KIRC,

PAAD, UCS, THCA and SKCM in TCGA dataset. (b) Expression levels of the RCC1 gene by different pathological stages of BLCA, BRCA, OV, CHOL, DLBC, ESCA, HNSC, LUAD, LUSC, CESC, READ, STAD, TGCT and UCEC.

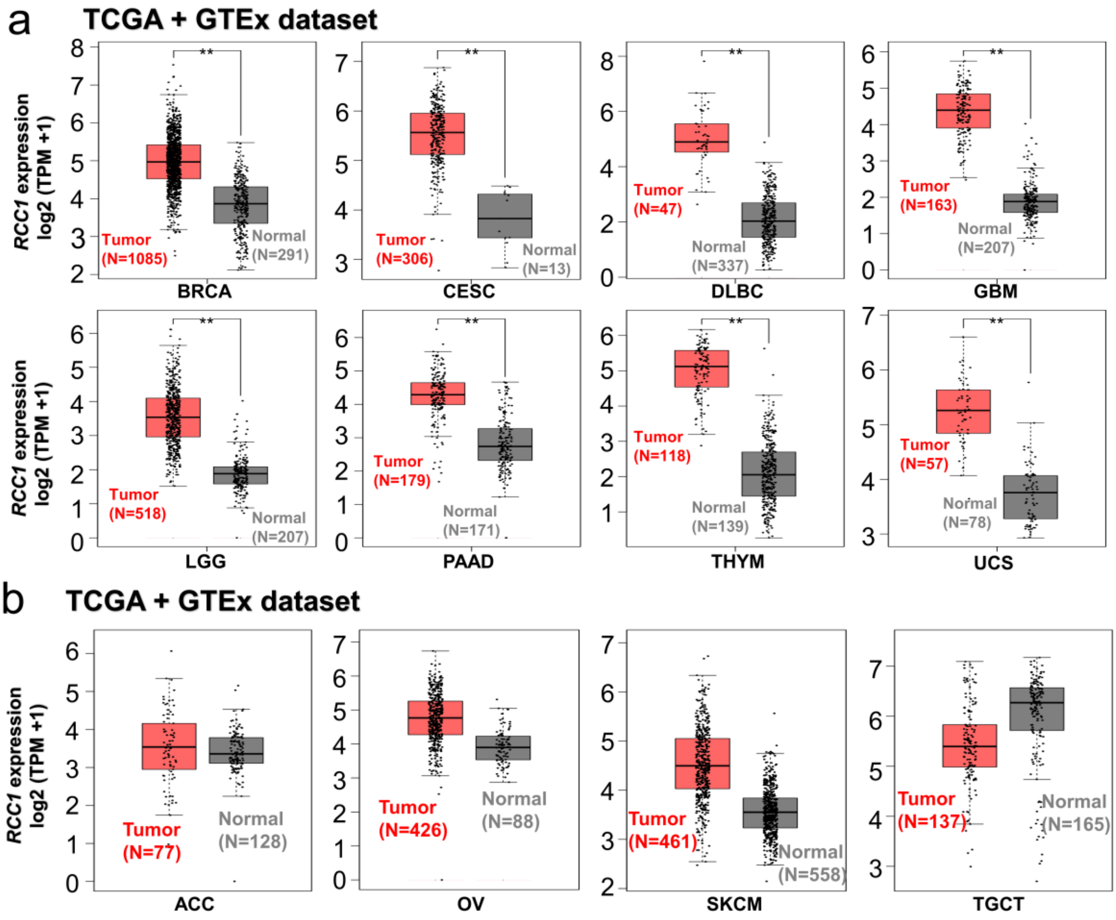

Supplementary Figure S3. Expression level of RCC1 gene in different tumors based on the combination of TCGA and GTEx datasets. (a) The expression of RCC1 in BRCA, CESC, DLBC, GBM, LGG, PAAD, THYM and UCS based on the combination of TCGA and GTEx datasets. ** *P* <0.01. (b) The expression of RCC1 in ACC, OV, SKCM and TGCT based on the combination of TCGA and GTEx datasets.

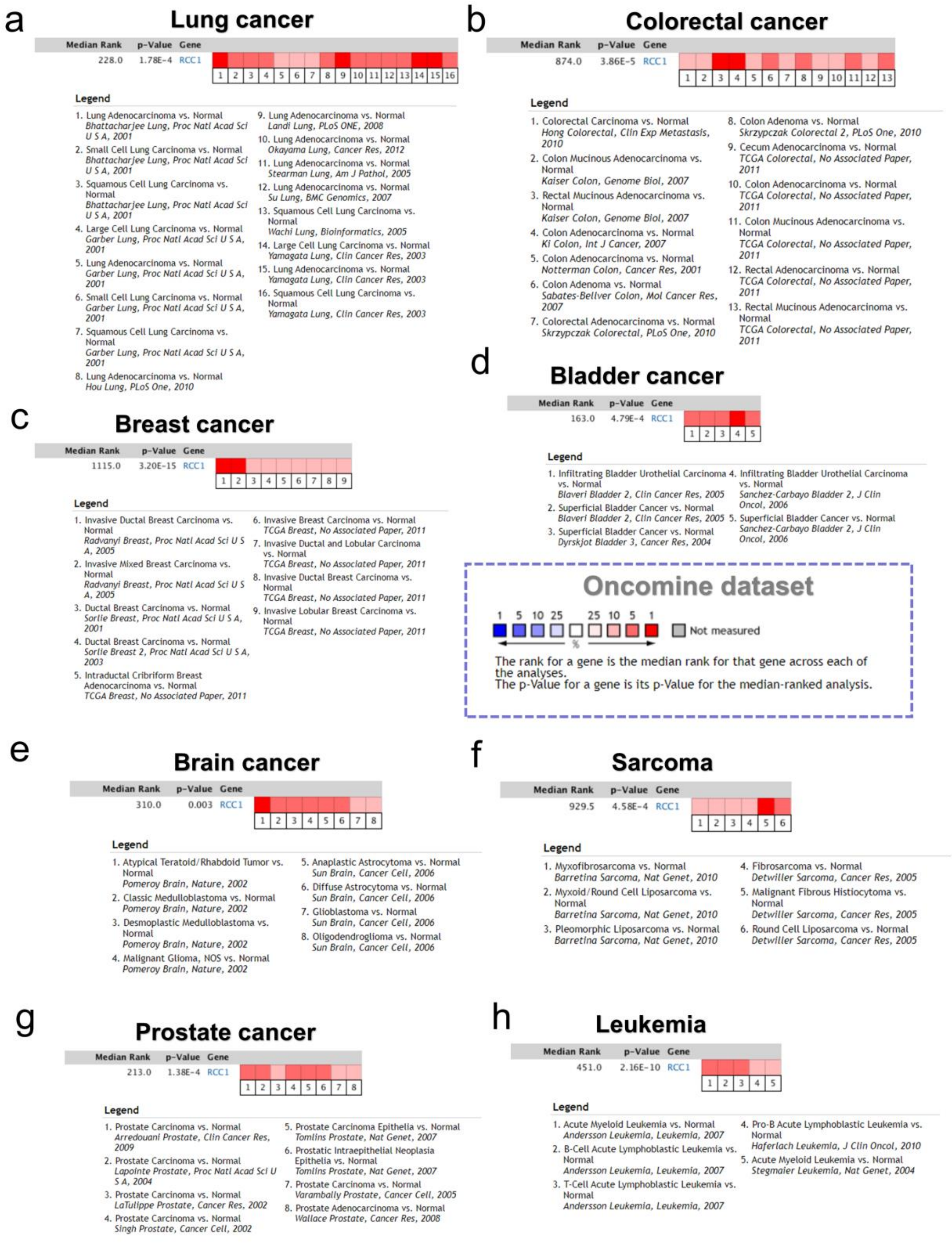

Supplementary Figure S4. Pooled analysis on the RCC1 expression difference between normal and tumor tissues via the Oncomine database. (a) Lung cancer; (b) Colorectal cancer; (c) Breast cancer; (d) Bladder cancer; (e) Brain cancer; (f) Sarcoma; (g) Prostate cancer; (h) Leukemia.

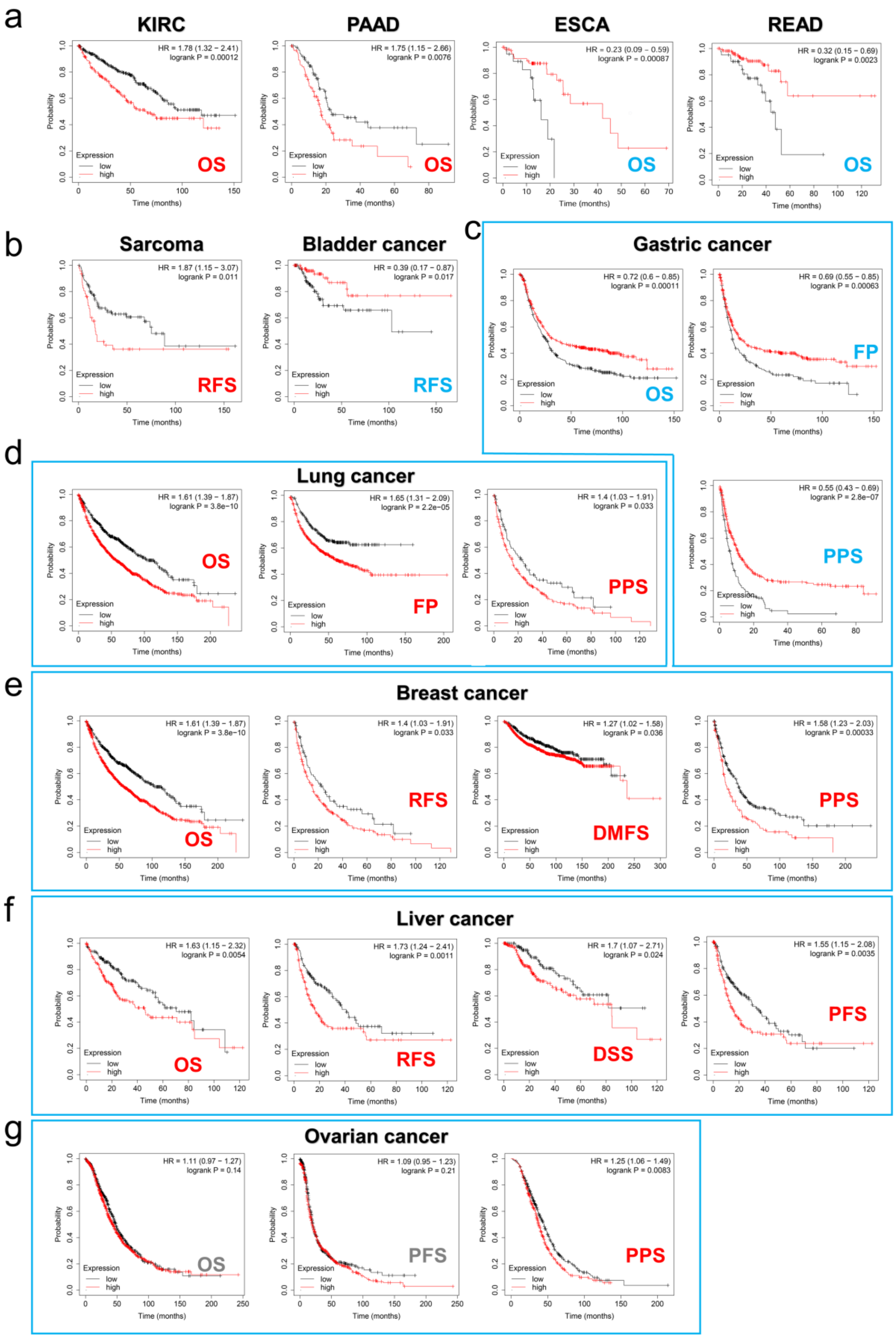

Supplementary Figure S5. Correlation between RCC1 gene expression and prognosis of different tumors based on the Kaplan-Meier plotter. (a) KIRC, PAAD,

ESCA and READ; (b) Sarcoma and bladder cancer; (c) Gastric cancer; (d) Lung cancer; (e) Breast cancer; (f) Liver cancer; (g) Ovarian cancer.

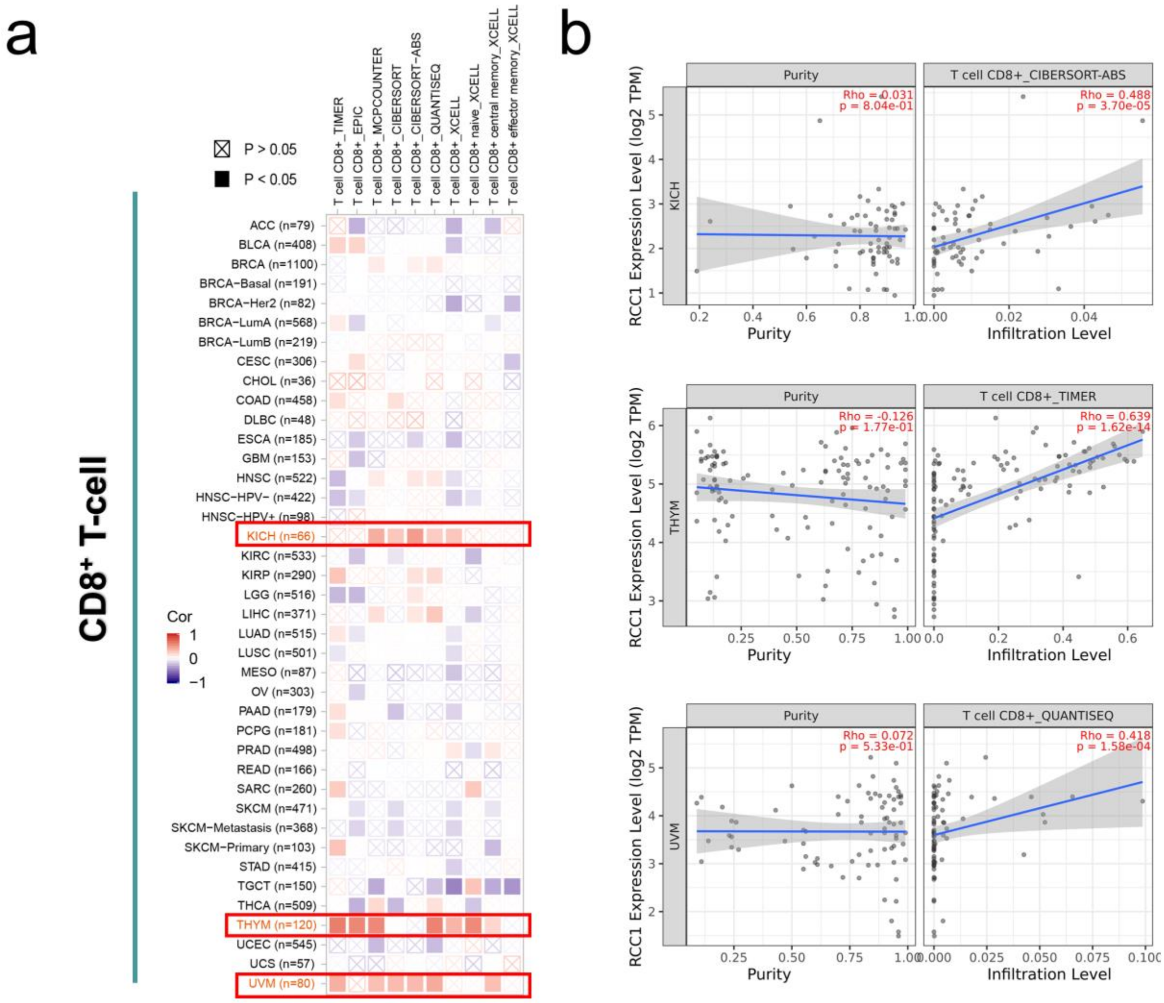

Supplementary Figure S6. Association between RCC1 expression and immune infiltration of CD8^{+} T cells in different tumors. The scatter plots of CD8^{+} T cells immune infiltration in different tumors were generated based on a certain algorithm.

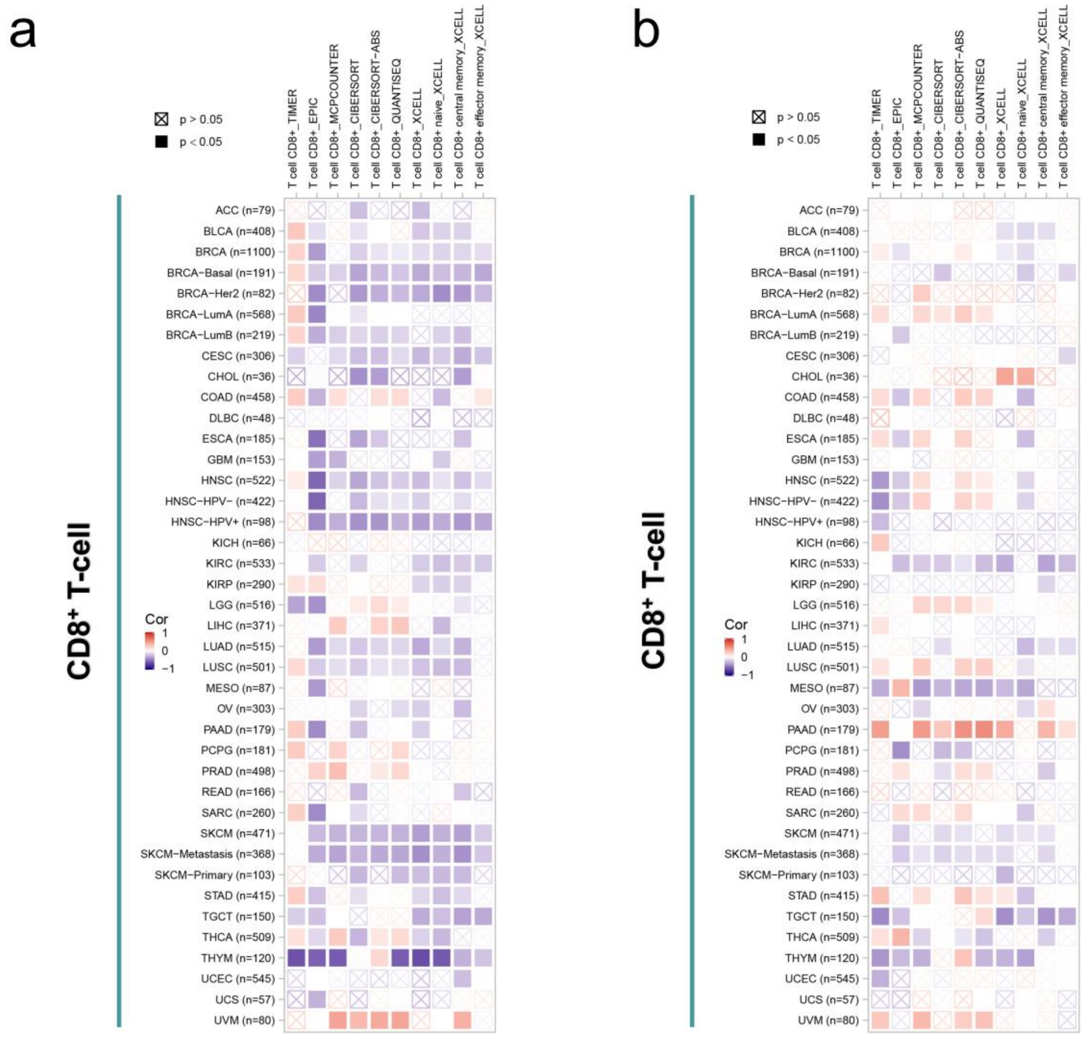

Supplementary Figure S7. Association between CD276 (a) and NRP1 (b) expression and immune infiltration of CD8^{+} T cells in different tumors.

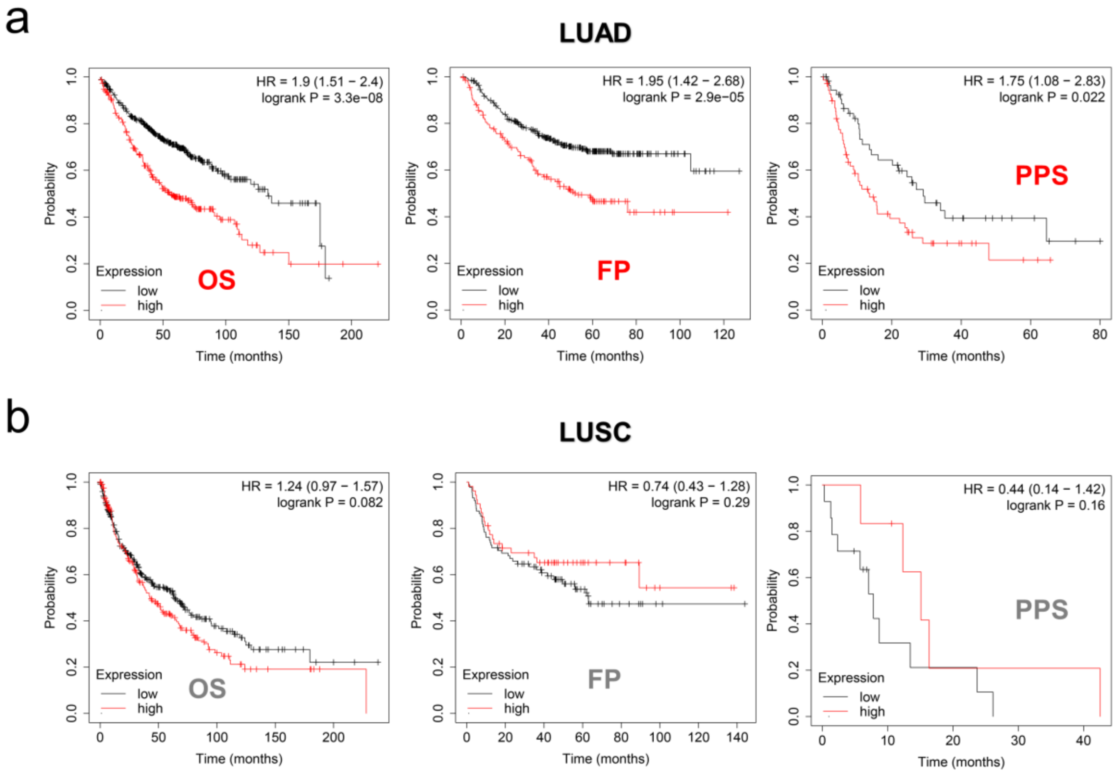

Supplementary Figure S8. Correlation between RCC1 gene expression and prognosis of LUAD/LUSC based on the Kaplan-Meier plotter.

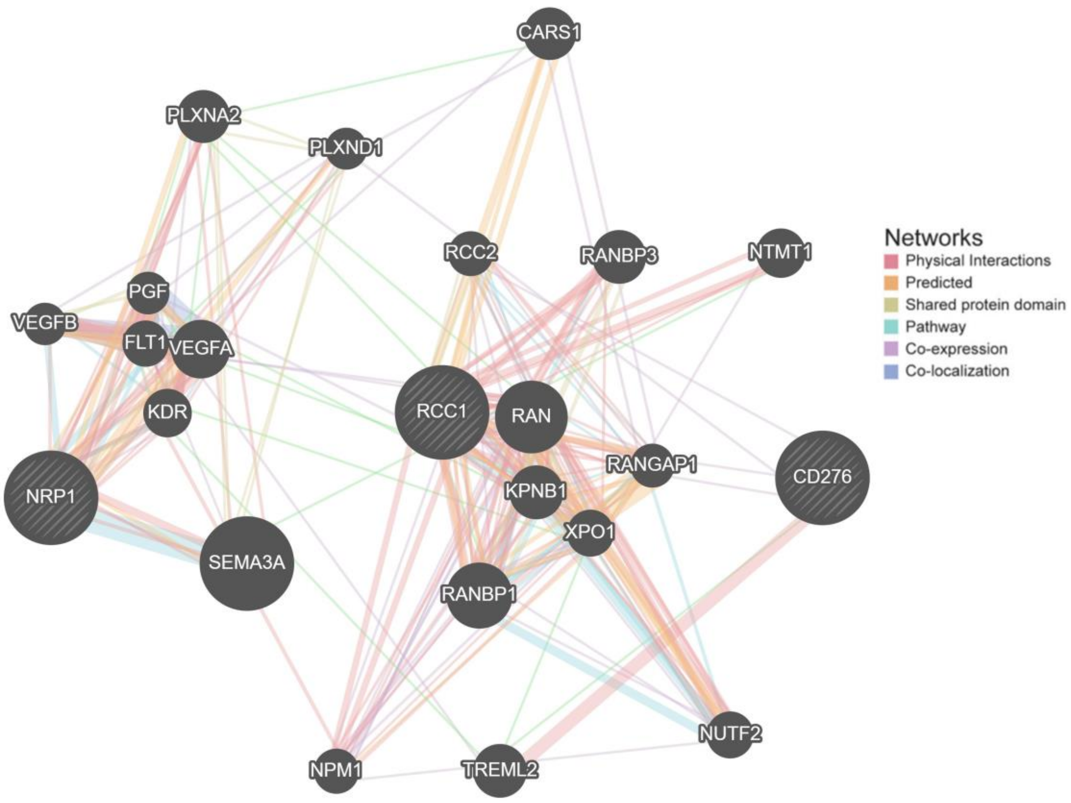

Supplementary Figure S9. Network of RCC1, CD276 and NRP1 was analyzed by GeneMANIA.

Supplementary Table S2. Correlation of RCC1 gene expression with RCC1 DNA methylation in multiple probes of different tumors.

Tumor Type	Promoter Region	Non-Promoter Region
BLCA	NC	NC
BRCA	NC	NC
CESC	NC&PC	NC&PC
CHOL	No correlation	NC
COAD	NC	NC&PC
DLBC	NC	NC&PC
ESCA	NC	NC&PC
GBM	No correlation	NC&PC
HNSC	No correlation	NC&PC
KICH	No correlation	NC
KIRC	No correlation	NC
KIRP	No correlation	NC&PC
LAML	No correlation	NC&PC

LGG	NC&PC	NC&PC
LIHC	NC	NC
LUAD	NC	NC
LUSC	No correlation	NC&PC
MESO	NC	NC
OV	NC	NC
PAAD	No correlation	NC
PCPG	NC	NC&PC
PRAD	No correlation	NC&PC
READ	NC	NC
SARC	NC	NC&PC
SKCM	NC	NC&PC
STAD	No correlation	NC&PC
TGCT	NC	NC&PC
THCA	NC	NC&PC
THYM	No correlation	NC&PC
UCEC	NC&PC	NC&PC
UVM	NC	NC&PC

NC, Negative correlation; PC, Positive correlation; BLCA, Bladder Urothelial Carcinoma; BRCA, Breast invasive carcinoma; CESC, Cervical squamous cell carcinoma and endocervical adenocarcinoma; CHOL, Cholangiocarcinoma; COAD, Colon adenocarcinoma; DLBC, Lymphoid Neoplasm Diffuse Large B-cell Lymphoma; ESCA, Esophageal carcinoma; GBM, Glioblastoma multiforme; HNSC, Head and Neck squamous cell carcinoma; KICH, Kidney Chromophobe; KIRC, Kidney renal clear cell carcinoma; KIRP, Kidney renal papillary cell carcinoma; LAML, Acute Myeloid Leukemia; LGG, Brain Lower Grade Glioma; LIHC, Liver hepatocellular carcinoma; LUAD, Lung adenocarcinoma; LUSC, Lung squamous cell carcinoma; MESO, Mesothelioma; OV, Ovarian serous cystadenocarcinoma; PAAD, Pancreatic adenocarcinoma; PCPG, Pheochromocytoma and Paraganglioma; PRAD, Prostate adenocarcinoma; READ, Rectum adenocarcinoma; SARC, Sarcoma; SKCM, Skin Cutaneous Melanoma; STAD, Stomach adenocarcinoma; TGCT, Testicular Germ Cell Tumors; THCA, Thyroid carcinoma; THYM, Thymoma; UCEC, Uterine Corpus Endometrial Carcinoma; UVM, Uveal Melanoma.
